# Vitamin D sufficiency and its relationship with muscle health across the menopausal transition and aging: Finnish cohorts of middle-aged women and older women and men

**DOI:** 10.1038/s41430-025-01610-4

**Published:** 2025-04-02

**Authors:** Satoshi Fujita, Hannamari Lankila, Kaisa Koivunen, Matti Hakamäki, Sarianna Sipilä, Erja Portegijs, Taina Rantanen, Eija K. Laakkonen

**Affiliations:** 1 Faculty of Sport and Health Science, Ritsumeikan University, Kusatsu, Japan; 2 Gerontology Research Center and Faculty of Sport and Health Sciences, University of Jyväskylä, Jyväskylä, Finland; 3 Jamk University of Applied Sciences, Jyväskylä, Finland; 4 Center of Human Movement Sciences, University Medical Center Groningen, University of Groningen, Groningen, Netherlands

**Keywords:** Biomarkers, Risk factors

## Abstract

**Background:**

Finland’s national vitamin D fortification policy has significantly improved the population’s vitamin D sufficiency. This study investigates the association between serum vitamin D concentration and muscle health, considering the impact of menopause and aging in Finnish cohorts.

**Methods:**

The study comprised two cohorts: 237 middle-aged women (aged 47–55 years) from the Estrogenic Regulation of Muscle Apoptosis (ERMA) study and its follow-up, and 908 older adults (aged 75, 80, and 85 years) from the Active Aging (AGNES) study. Vitamin D concentration was assessed through serum 25-hydroxyvitamin D (25(OH)D) concentrations, alongside measurements of muscle mass and function.

**Results:**

High concentrations of 25(OH)D were observed across both cohorts, aligning with Finland’s fortification efforts. Furthermore, no significant correlations were found between 25(OH)D concentrations and indicators of muscle mass or function in either age group. Notably, middle-aged women in menopausal transition exhibited a slight increase in 25(OH)D concentrations, yet this did not translate into improved muscle outcomes. Similarly, older adults demonstrated sufficient 25(OH)D concentrations without a corresponding enhancement in muscle health.

**Conclusions:**

The findings indicate that, within the context of Finland’s vitamin D fortification program, serum 25(OH)D sufficiency does not directly correlate with better muscle mass or function among middle-aged and older Finnish populations. These results suggest a need for a broader approach to sarcopenia prevention, incorporating factors beyond vitamin D sufficiency. Further research is warranted to explore the multifactorial nature of muscle health during aging and the menopausal transition, to develop targeted interventions for sarcopenia prevention.

## Introduction

Aging diminishes muscle mass and function, elevating the risk of falls, fractures, and metabolic disorders [1]. Studies even in middle-aged individuals indicate that reduced muscle mass contributes to worsened cholesterol profiles and insulin resistance [2]. Notably, a decade-long study on over 2000 adults found that higher skeletal muscle mass (SMM) significantly lowers cardiovascular disease (CVD) risk [3], highlighting its predictive value for CVD outcomes.

Research has shown a decline in lean mass between peri- and early postmenopause, with Juppi et al. reporting reductions of 0.5–1.5% during this transition, as measured by Dual-energy X-ray Absorptiometry (DXA) and Computed Tomography (CT) imaging [4]. The findings underscore the significance of hormonal shifts during menopause in reducing lean and muscle mass, distinct from aging’s impact. Specifically, cellular apoptosis pathways like heat-shock proteins, cell death receptor ligands, and microRNAs targeting apoptosis are primary suspects in muscle decline due to estrogen deficiency [5, 6]. Yet, whether the decrease in protein synthesis or heightened proteolysis due to hormonal changes drives this loss remains debated [6].

Vitamin D plays a critical role in muscle growth, development [7], and contraction [8]. Insufficient vitamin D concentration can impair muscle cell function [9], leading to reduced muscle mass and power, particularly in the lower limbs [10, 11]. This deficiency is also associated with diminished physical capabilities and a higher risk of falls, especially in the elderly [12]. Aging further impacts the synthesis of active vitamin D, exacerbating these effects [13]. Research links low 25-hydroxyvitamin D (25(OH)D) concentration in menopausal women to muscle mass reduction, independent of factors like age and lifestyle [14]. Studies confirm that vitamin D deficiency, particularly in menopausal women, is associated with muscle strength decline and increased sarcopenia risk [15]. Furthermore, vitamin D supplementation in postmenopausal women has been shown to enhance muscle strength and prevent lean mass loss [16], although no previous study has investigated the anabolic effect of vitamin D supplementation during menopausal transition. These results underscore the importance of adequate vitamin D for muscle preservation among middle-aged and older women.

Launched in 2003, Finland’s fortification policy, aimed at combating vitamin D deficiency due to limited sunlight, has markedly enhanced the population’s vitamin D status by fortifying milk products and fat spreads with vitamin D3 and promoting oral supplementation in darker months [17, 18]. This initiative has significantly raised vitamin D intake from an average of 5 µg/day to 17 µg/day in men and 3 µg/day to 12 µg/day in women, resulting in an average serum 25(OH)D concentration increase of 17 nmol/L [19]. While no direct comparison exists regarding sarcopenia rates before and after Finland’s fortification policy, the notable improvement in vitamin D status implies a strong potential to reduce muscle mass and function decline in menopausal and post-menopausal women.

The purpose of this study is to assess the association between serum 25(OH)D concentrations and SMM and function in middle-aged and older populations in Finland, considering the impact of menopausal status and lifestyle factors. The study aims to provide insights into the potential role of vitamin D in preserving muscle health against age-related decline and the impact of lifestyle factors such as diet, supplement use, physical activity, and obesity on vitamin D status within the Finnish context.

## Subjects and methods

### Study design

This study uses the data and blood samples from two observational datasets comprising middle-aged women and older women and men age cohorts. The middle-aged women cohort is part of the Estrogenic Regulation of Muscle Apoptosis (ERMA) study and its four-year follow-up Estrogen, MicroRNAs and the Risk of Metabolic Dysfunction (EsmiRs) study (dataset: doi.10.17011/jyx/dataset/83491) representing 47–55-years old white Finnish women. The older cohort is part of the Active aging – resilience and external support as modifiers of the disablement outcome (AGNES) study [20] including three age cohorts (75, 80, and 85 years) of white Finnish women and men living independently in the city of Jyväskylä in Central Finland (dataset: doi.10.17011/jyx/dataset/83811). The ERMA study was approved by the Ethics Committee of the Central Finland Health Care District in 2014 (K-S shp Dnro U/2014). Participants provided two separate signed consents: initial consent for the use of prequestionnaire data in research and publications (phase one) and additional informed consent before laboratory examinations, detailing potential risks and benefits and authorizing data use for research and publications (phase two) [21]. The AGNES study was approved by the Ethics Committee of the Central Finland Health Care District on August 23, 2017. Participants provided written informed consent before the home interview, with separate approvals for research center assessments, patient record use, and future contact. The study adhered to the Declaration of Helsinki [20].

The ERMA study began in December 2014 [21] with subsequent phases including EsmiRs starting November 2018 [22]. The ERMA study initially included 1393 women, with 1158 participating in later phases. The sample and contact information were sourced as a random sample of the target population from the Population Information System managed by the Population Register Center (http://vrk.fi/en). To minimize self-exclusion and ensure a representative sample for the study’s initial phase, the invitation letter did not mention exclusion criteria. Exclusion criteria included significant health conditions or medication use affecting hormonal or inflammatory statuses. For EsmiRs, 811 were invited, with 494 completing questionnaires and 304 undergoing lab tests. The AGNES study was collected between September 2017 to December 2018 [23]. It targeted 2791 individuals, with 1021 completing surveys or interviews and 910 participating in physiological tests. All studies adhered to the Declaration of Helsinki, with participant consent and ethical approval (ERMA 8U/2014, EsmiRs 9U/2018, AGNES August 23, 2017). The current analysis focused on participants with available 25(OH)D blood samples and at least one physiological test, including 237 middle-aged women and 908 older individuals (56.8% women).

### Measurement of vitamin D concentration, vitamin D sufficiency groups and use of vitamin D supplements

Fasting (middle-aged women cohort) and non-fasting (older women and men cohort) blood samples were taken from the antecubital vein. Serum was collected by standard procedures, aliquoted and stored at −80 °C or immediately used for biomarker assessments. For the current study, 25(OH)D concentration was measured by two clinical laboratories: Vita Laboratoriot Oy (Helsinki, Finland) using Elecsys Vitamin D total II electrochemiluminescence binding assay (Roche Diagnostics, Finland) and Synlab Suomi (Espoo, Finland) using ARCHITECT chemiluminescent microparticle immunoassay (Abbot architect, USA). Serum 25(OH)D cut-offs are defined by the Institute of Medicine (IOM) as follows: deficiency (<30 nmol/L), inadequacy (30 to <50 nmol/L), sufficiency (≥50 nmol/L), and excessive (>125 nmol/L), based on the 2011 guidelines [24].

In the middle-aged women cohort, participants self-reported the use of any supplements, number of supplements and type or brand names of the used supplements. This data was used to construct the following variables: use of any supplement (yes/no), number of supplements used (none, 1–2, 3–5, ≥6), and use of vitamin D supplements (yes/no). In the older women and men cohort, participants self-reported the use of medication prescribed by a medical doctor, but the use of self-prescribed supplements was not asked. Of the reported medications, a variable use of vitamin D medication was constructed (yes/no).

### Physiological tests

Detailed procedures for physiological tests are described in the Supplemental Information. Briefly, body composition was assessed using DXA (LUNAR Prodigy; GE Healthcare, Chicago, IL) for middle-aged women and BIA (InBody 720, Biospace, Seoul, Korea) for older women and men, analyzing total (LBM) and appendicular lean body mass (summed lean mass of arms and legs, ALM) as SMM proxies. Similarly, BIA provided skeletal (SM) and soft lean mass estimates (LBM).

Both cohorts underwent knee extension (KE) and handgrip force (HG) evaluations using a custom dynamometer chair (Good Strength; Metitur Oy, Palokka, Finland), with measurements taken at specified limb angles and positions [20, 23, 25, 26]. The middle-aged women’s cohort also had vertical jump height assessed via a contact mat, calculating elevation from flight time [25]. For all performance tests, the best of three to five maximal efforts was recorded.

### Descriptive and other variables

In both cohorts, socioeconomic status data was obtained via questionnaires, with education categorized as ≤secondary or ≥tertiary, and smoking status as never or ever (including current/past smokers). For the middle-aged women cohort, menopausal status was assessed using menstrual diaries and follicle-stimulating hormone (FSH) measurements at the ERMA baseline [21], and by questionnaires and FSH at the EsmiRs [22]. This informed a menopausal transition variable comprising three groups: transition (from pre-/perimenopausal to postmenopausal), PRE/PERI (consistent pre-/perimenopausal), and POST (consistent postmenopausal). The Executive Summary of the Stages of Reproductive Aging criteria for defining menopausal status is based on increasing variability (in perimenopause) and finally the absence of menstrual bleeding for 12 months (in postmenopause), as well as elevated FSH levels in two consecutive measurements [27]. The ERMA study used self-reported menstrual cycles and two FSH measurements to confirm postmenopause. When tracking menstrual bleeding was not possible, i.e., for women using progesterone-releasing intrauterine devices or those who had undergone hysterectomy, FSH levels were the primary criterion.

In the middle-aged women cohort, physical activity was measured using the SR-PA L7 scale, categorizing activities into low, medium, and high levels based on daily activities to competitive sports [28]. Longitudinal activity groups were established based on changes in these levels over time. Leisure-time activity was evaluated through a questionnaire assessing frequency, intensity, duration, and commuting time, from which MET hours per day for leisure activity were calculated [29]. Physical activity of older women and men was categorized into three levels using the activity scale with six options was used, omitting the lowest activity level due to its rare selection in past studies [30]. In the ERMA and EsmiRs studies, participants completed the main questionnaires at home before visiting the research center, where staff checked for missing responses. In the AGNES study, questionnaires were completed during face-to-face interviews at participants’ homes or the research center. Trained research staff conducted these interviews to ensure consistency and minimize missing data. In both cohorts, body mass and height were measured, and body mass index (BMI; kg/m^2^) was calculated. Participants were categorized into normal (BMI ≤ 24.9), overweight (BMI 25–29.9), and obesity (BMI ≥ 30). Longitudinally, BMI categories were defined based on changes toward normal weight, obesity, or severe obesity.

Seasonality was recorded at blood sampling and categorized into winter (December, January, and February), spring (March, April, and May), summer (June, July, and August), and autumn (September, October and November), with winter and autumn as dark seasons due to limited sunlight. In the cohort of middle-aged women, sampled twice, seasons were classified into light season (spring or summer at both timepoints), towards lightness (baseline sampling at winter or autumn and follow-up sampling at spring or summer), towards darkness (baseline sampling at spring or summer and follow-up sampling at winter or autumn), or dark season (winter or autumn at both timepoints) based on the sampling timeline.

### Statistics

Descriptive statistics including frequencies, means, and standard deviations were used to describe the study cohorts. Univariate group comparisons were performed using t-tests or general linear models (GLM). Repeated measurement data structure of the middle-aged women cohort was taken into account in the analysis. Potential confounders identified by the literature were age, sex, season, education, smoking, physical activity, and BMI, influencing 25(OH)D concentrations. Univariate models linked sex, menopausal status, physical activity, and BMI to 25(OH)D concentrations. Adjustments for body dimensions used body mass and height instead of BMI. Linear mixed-effect models for middle-aged women included time (0 = baseline, 1 = follow-up), vitamin D, menopausal status, body mass, height, and physical activity, excluding sex and age due to uniformity. Linear regression for older cohorts used 25(OH)D concentration as a predictor with confounders like sex, body mass, height, physical activity, and mean-centered age; menopausal status was omitted as participants were postmenopausal. Sex-stratified analyses, showing no significant differences, led to a combined analysis with sex as a covariate. The normality of residuals and other model assumptions were evaluated by graphical methods before accepting the results. All statistical analyses were performed with IBM SPSS Statistics software version 28 (Chicago, IL, US), and *p* < 0.05 was considered statistically significant.

## Results

Based on the measured 25(OH)D concentrations, 79–90% of participants were classified as having “sufficient” 25(OH)D levels (Table 1). Older men showed the highest deficiency (2%) and insufficiency (8%), with excessive 25(OH)D concentrations most common among them (10%) and least common in middle-aged women at baseline (6%).

**Table 1:** Vitamin D sufficiency levels and supplement use in middle-aged women cohort and older women and men cohorts.

	Middle-aged women cohort				Older cohort			
	Baseline (*n* = 237)		Follow-up (*n* = 237)		Women (*n* = 516)		Men (*n* = 392)	
	% (*n*)	25(OH)D^a^ [nmol/l]	% (*n*)	25(OH)D^a^ [nmol/l]	% (*n*)	25(OH)D^a^ [nmol/l]	% (*n*)	25(OH)D^a^ [nmol/l]
Vitamin D sufficiency groups								
Deficiency (<30 nmol/l)	–	–	–	–	0.4 (2)	30.0 ± 0.0	2.3 (9)	24.6 ± 4.7
Inadequacy (30 to <50 nmol/l)	4.6 (11)	42.5 ± 5.4	6.3 (15)	42.6 ± 4.1	7.8 (40)	43.2 ± 5.0	8.4 (33)	41.3 ± 5.5
Sufficiency (≥50–125 nmol/l)	89.5 (212)	81.0 ± 17.21	84.0 (199)	83.6 ± 18.7	83.9 (433)	84.6 ± 17.7	79.1 (310)	79.0 ± 19.1
Excessive (>125 nmol/l)	5.9 (14)	140.8 ± 20.2	9.7 (23)	148.3 ± 27.5	7.9 (41)	155.7 ± 33.9	10.2 (40)	145.1 ± 17.8
Use of vitamin D medication^b^								
Non-user					84.3 (435)	85.7 ± 30.5	93.1 (365)	80.1 ± 30.6
User					15.7 (81)	92.7 ± 26.3	6.9 (27)	98.0 ± 30.0
Use of vitamin D supplement^c^								
Non-user	20.3 (48)^e^	68.2 ± 16.6	27.0 (64)^e^	68.3 ± 19.9				
User	79.7 (189)	86.4 ± 23.9	73.4 (173)	94.4 ± 29.3				
Use of any supplements^c^								
Non-user	12.7 (30)	66.2 ± 15.5	19.8 (47)^d^	67.5 ± 21.4				
User	87.3 (207)	85.1 ± 23.8	80.2 (190)	92.2 ± 29.1				
Number of supplements used^c^								
None	12.1 (30)^e^	66.7 ± 15.5	19.8 (47)^e^	67.6 ± 21.4				
1–2	53.2 (126)	82.0 ± 19.3	47.2 (112)	85.5 ± 23.9				
3–5	30.0 (71)	86.0 ± 24.2	30.0 (71)	99.4 ± 28.3				
6 or more	4.2 (10)	119.4 ± 42.8	3.0 (7)	126.4 ± 63.5				

^a^Values are mean ± standard deviation.

^b^Data not available for the middle-aged women cohort.

^c^Data not available for the older cohort.

^d^Three unclear answers were coded as non-user.

^e^One missing answer was coded as non-user.

The self-reported use of any type of supplements including those containing vitamin D, appeared to be frequent among middle-aged women. At baseline nearly 80% and at follow-up 73% reported using vitamin D-containing supplements. The corresponding numbers for the use of any supplements were 87% and 85%, respectively. Of the older women and men cohort, only the use of medication prescribed by physicians was assessed, thus numbers cannot be compared between cohorts. Of the older women, 16%, and of the older men, 7% reported using vitamin D-containing drugs.

In the middle-aged women cohort, 25(OH)D concentrations were generally high and significantly higher at follow-up timepoint than at baseline (82.8 ± 23.8 vs. 87.3 ± 29.4 nmol/l, *p* < 0.001, Table 2). Group variables were constructed from the potential effectors of the 25(OH)D concentrations and presented in Table 2.

**Table 2:** Differences in 25(OH)D (nmol/l) concentrations between baseline and follow-up timepoints in middle-aged women cohort study stratified by potential effectors.

Group variables	Baseline mean ± SD	Follow-up mean ± SD	*p*-value within-subjects effects (time)	*p*-value between-subjects effects (group)	*p*-value for time x group interaction
All	82.8 ± 23.8 (*n* = 237)	87.3 ± 29.4 (*n* = 237)	**<0.001**		
Education at baseline			**<0.001**	0.551	0.074
At most secondary	84.6 ± 25.9 (*n* = 136)	87.2 ± 31.4 (*n* = 136)			
At least tertiary	80.3 ± 20.4 (*n* = 101)	87.5 ± 26.7 (*n* = 101)			
Smoking status			**0.004**	0.922	0.338
Never smoker	82.2 ± 23.1 (*n* = 163)	87.6 ± 27.7 (*n* = 163)			
Ever smoker	83.9 ± 25.3 (*n* = 74)	86.6 ± 33.2 (*n* = 74)			
Menopausal groups			**0.002**	**0.002**	0.811
Transition	78.6 ± 21.9 (*n* = 135)	83.1 ± 25.7 (*n* = 135)		0.001 comp. to POST	
PRE/PERI	83.7 ± 21.8 (*n* = 45)	87.9 ± 27.7 (*n* = 45)			
POST	91.8 ± 27.1 (*n* = 57)	97.7 ± 36.3 (*n* = 57)			
Physical activity			0.209	**0.033**	0.152
Low	76.9 ± 19.7 (n = 18)	73.2 ± 24.9 (*n* = 18)		0.083 comp. to high	
Medium	79.4 ± 24.0 (*n* = 102)	85.5 ± 28.3 (*n* = 102)			
High	86.6 ± 23.7 (*n* = 117)	91.130.4 (*n* = 117)			
Body mass index			**<0.001**	**0.023**	0.331
Normal	86.9 ± 26.6 (*n* = 105)	93.0 ± 32.2 (*n* = 105)		0.024 comp. to with obesity	
Overweight	79.5 ± 20.5 (*n* = 94)	81.7 ± 24.7 (*n* = 94)			
Obesity	79.3 ± 21.8 (*n* = 38)	85.6 ± 30.1 (*n* = 38)			
Diet; use of fatty fish			**0.001**	0.677	0.761
Rarely (at most monthly)	81.4 ± 25.9 (*n* = 59)	84.4 ± 33.6 (*n* = 59)			
Once a week	82.7 ± 18.4 (*n* = 98)	87.3 ± 23.5 (*n* = 98)			
Often (at least twice a week)	83.8 ± 27.8 (*n* = 80)	89.5 ± 32.7 (*n* = 80)			
Diet; use of (fortified) milk-products			**<0.001**	0.684	0.303
Rarely (at most once a week)	80.8 ± 24.3 (*n* = 76)	87.3 ± 33.5 (*n* = 76)			
Regularly (at least twice a week)	83.7 ± 23.5 (*n* = 161)	87.3 ± 27.4 (*n* = 161)			
Season at time of blood sampling			**0.023**	0.425	0.963
Light season	80.8 ± 26.7 (*n* = 95)	84.5 ± 33.4 (*n* = 95)			
Toward lightness	80.4 ± 16.8 (*n* = 8)	85.5 ± 26.8 (*n* = 8)			
Toward darkness	81.4 ± 22.5 (*n* = 53)	86.7 ± 25.2 (*n* = 53)			
Dark season	86.2 ± 21.3 (*n* = 81)	91.2 ± 27.2 (*n* = 81)			

Significant *p*-values are bolded.

Women transitioning from pre- or perimenopause to postmenopause showed a smaller increase in 25(OH)D concentrations compared to those who were postmenopausal at both timepoints (4.5 vs. 5.9 units, *p* = 0.001). Physical activity level was also associated significantly with 25(OH)D concentration (*p* = 0.033). Women belonging to the normal body mass group had higher 25(OH)D concentrations than women belonging to the group with obesity (*p* = 0.024). In older women and men cohort, 25(OH)D concentrations were higher among women than men (86.8 ± 30.0 vs. 81.3 ± 30.9, *p* = 0.007, Table 3). Potential effectors that may influence 25(OH)D concentrations are presented as group variables in Table 3. Only BMI had a significant group difference (*p* = 0.004) while all others reproduced the sex difference in 25(OH)D concentrations.

**Table 3:** Differences in 25(OH)D (nmol/l) concentrations between women and men groups of older cohort data stratified by potential effectors.

Group variables	Women mean ± SD	Men mean ± SD	*p*-value between-subjects effects (sex)	*p*-value between-subjects effects (group)
All	86.8 ± 30.0 (*n* = 516)	81.3 ± 30.9 (*n* = 392)	**0.007**	
Education at baseline			**0.007**	0.836
At most secondary	86.8 ± 29.7 (*n* = 309)	80.6 ± 30.4 (*n* = 207)		
A least tertiary	86.7 ± 30.6 (*n* = 203)	81.8 ± 31.3 (*n* = 182)		
Smoking status			**0.030**	0.975
Never smoker	86.3 ± 29.8 (*n* = 424)	82.4 ± 29.8 (*n* = 202)		
Ever smoker	88.1 ± 29.8 (*n* = 72)	81.0 ± 32.2 (*n* = 171)		
Physical activity			**0.002**	0.279
Low level	83.3 ± 31.8 (*n* = 73)	76.1 ± 33.6 (*n* = 39)		
Medium level	87.7 ± 30.4 (*n* = 385)	80.6 ± 30.9 (*n* = 277)		
High level	85.7 ± 25.6 (*n* = 50)	85.2 ± 28.6 (*n* = 69)		
Body mass index			**0.002**	**0.004**
Normal	88.68 ± 25.4 (*n* = 122)	86.1 ± 31.3 (*n* = 110=		
Overweight	89.0 ± 31.7 (*n* = 225)	80.7 ± 30.0 (*n* = 180)		
Obesity	81.6 ± 25.8 (*n* = 145)	74.6 ± 29.4 (*n* = 59)		
Season at time of blood sampling			**0.010**	0.197
Spring	89.5 ± 29.8 (*n* = 122)	78.8 ± 30.7 (*n* = 108)		
Summer	83.2 ± 28.0 (*n* = 56)	76.6 ± 27.5 (*n* = 46)		
Autumn	86.9 ± 29.9 (*n* = 245)	84.6 ± 32.1 (*n* = 179)		
Winter	82.8 ± 23.4 (*n* = 92)	79.4 ± 29.5 (*n* = 59)		

Significant *p*-values are bolded.

Associations of 25(OH)D concentration with proxies of SMM and KE, HG, and VJH were analyzed using linear mixed-effect models for the middle-aged women cohort (Table 4) and linear regression models for older women and men cohort (Table 5). The 25(OH)D concentration had a negligible effect with a non-significant regression coefficient of −0.034 to 0.082 in models constructed with data from middle-aged women and <0.001 to 0.106 in models constructed with data from older women and men when models were controlled for potential confounding factors.

**Table 4:** Effect estimates for physiological variables in the longitudinal middle-aged women cohort study.

	ALM [kg]	LBM [kg]	KE [N]	HG [N]	VJH [cm]
	B [95% CI]	B [95% CI]	B [95% CI]	B [95% CI]	B [95% CI]
Intercept	−12.56*** [−18.12, −7.01]	−13.88* [−25.06, −2.70]	37.72 [−316.18, 391.62]	−106.91 [−329.89, 116.07]	9.09 [−5.13, 23.32]
*Main effects*					
25(OH)D [nmol/l]	0.001 [−0.004, 0.005]	0.001 [−0.006, 0.008]	−0.034 [−0.35, 0.28]	0.082 [−0.11, 0.28]	−0.005 [−0.02, 0.01]
Menopausal group					
Transition (ref)					
PRE-PRE	0.58* [0.10, 1.05]	1.26** [0.30, 2.21]	21.9 [−7.74, 51.57]	15.51 [−3.30, 34.32]	1.66** [0.47, 2.86]
POST-POST	−0.11 [−0.54, 0.32]	−0.25 [−1.13, 0.62]	−6.86 [−34.63, 20.92]	−13.30 [−30.76, 4.16)	−0.98 [−2.08, 0.13]
Body mass [kg]	0.08*** [0.07, 0.10]	0.21*** [0.18, 0.24]	1.40** [0.39, 2.41]	0.19 [−0.43, 0.81]	−0.18*** [−0.22, −0.14]
Body height [m]	14.70*** [11.24, 18.16]	24.43*** [17.49, 31.69]	180.91 [−41.13, 402.95]	245.83*** [106.73, 384.93]	13.14** [4.26, 22.03]
PA [MET-h/d]	0.06*** [0.03, 0.09]	0.12*** [0.07, 0.16]	4.01*** [2.12, 6.03]	0.45 [−0.77, 1.67]	0.11** [0.04, 0.19]
Time (baseline-follow-up)	−0.46***[−0.34, −0.57]	−0.67*** [−0.49, −0.86]	−14.81*** [−6.18, −23.43]	8.39* [13.74, 3.04]	−1.04*** [0.71, 1.37]

*ALM* appendicular lean mass, *LBM* lean body mass, *KE* knee extension force, *HG* hand grip force, *VJH* vertical jumping height, *PA* physical activity.

^*^p ≤ 0.05; **p ≤ 0.01; ***p < 0.001.

**Table 5:** Effect estimates for physiological variables in the older women and men cohort study.

	SM [kg]	LBM [kg]	KE [N]	HG [N]
	B [SE]/β	B [SE]/β	B [SE]/β	B [SE]/β
(Constant)	(−25.33 [1.89])***	(−39.44 [2.95]) ***	(85.00 [89.63])	(−154.16 [60.78])*
25(OH)D [nmol/l]	<0.001 [0.002]/0.002	<0.001 [0.003]/0.000	0.024[0.101]/0.006	0.106 [0.068]/0.031
Sex (ref. men)	−3.65 [0.20]/−0.34***	−5.70 [0.31]/−0.32***	−110.29 [9.31]/−0.47***	−111.19 [6.32]/−0.55***
Body weight [kg]	0.16 [0.01]/0.37***	0.26 [0.01]/0.38***	1.41 [0.27]/0.16***	1.34 [0.18]/0.17***
Body height [m]	0.24 [0.01]/0.41***	0.41 [0.09]/0.42***	0.71 [0.54]/0.06	2.32 [0.37]/0.21***
PA (ref. high)				
medium	0.94 [0.18]/0.08***	1.39 [0.29]/0.07***	36.10 [8.74]/0.14***	18.60 [5.91]/0.08*
low	1.83 [0.26]/0.11***	2.56 [0.40]/0.10***	98.06 [12.21]/0.28***	45.10 [8.27]/0.15***
Age (mean centered)	−0.09 [0.02]/−0.06***	−0.10 [0.03]/−0.04***	−5.74 [0.86]/−0.18***	−4.72 [0.58]/−0.17***

*SM* skeletal muscle, *LBM* lean body mass, *KE* knee extension, *HG* hand grip, *PA* physical activity.

^*^p ≤ 0.05; ***p < 0.001.

## Discussion

This study explored the link between serum 25-hydroxycholecalciferol (25(OH)D) and muscle mass and function in Finnish middle-aged women and older adults. Findings revealed that none of the middle-aged women were vitamin D deficient, with the majority displaying sufficient 25(OH)D concentrations. A small fraction of the older cohort had vitamin D deficiency, though most had adequate levels. Notably, after adjusting for confounders, no significant correlation was found between 25(OH)D concentrations and muscle mass or function in any group, indicating that serum 25(OH)D is not related to the loss of muscle mass or function due to menopause or aging in these cohorts.

Aging leads to a 50% decrease in vitamin D biosynthesis, attributed to lower 7-dehydrocholesterol levels and reduced UV light response [13]. Studies have shown serum 25(OH)D concentrations are lower in postmenopausal than in premenopausal women [31]. Premenopausal women have higher serum 25(OH)D and vitamin D-binding protein (DBP) levels compared to postmenopausal women, with estradiol independently associated with DBP concentrations [31]. However, the precise connection between DBP and estrogen remains uncertain. In our study, no middle-aged subjects were “deficient” in vitamin D, with only 4–6% “insufficient”. Among the older cohort, 0.4% of women and 2.3% of men had insufficient 25(OH)D concentrations. These findings indicate that Finnish middle-aged and older individuals mostly maintained healthy serum 25(OH)D concentrations, and contrary to previous research [32], postmenopausal women had significantly higher 25(OH)D concentrations than pre- or perimenopausal women.

In this study we observed relatively high group mean concentrations of 25(OH)D ranging from 81 to 87 nmol/l and at group mean level being above 50 nmol/l threshold level even among those who reported not to use supplements, indicating that policy has been successful. An 11-year follow-up study (from 2000 to 2011) in Finland, showed serum 25(OH)D concentrations rose from 48 nmol/l to 65 nmol/l in men and from 48 nmol/l to 66 nmol/l in women, with the prevalence of levels below 50 nmol/l dropping from 55% to 9% in men and from 57% to 9% in women [33]. However, our research did not find a significant correlation between fortified milk intake and 25(OH)D concentrations in middle-aged women, suggesting the possible role of other fortified items. Furthermore, 70–80% of middle-aged women in the present study took supplements containing vitamin D, which may also have contributed to the increase in serum 25(OH)D and prevented the age-associated reduction in serum 25(OH)D. In the older cohort, we did not have information regarding the supplement use but only 16% of women and 7% of men reported use of vitamin D containing medication prescribed by a physician. Nevertheless, the serum 25(OH)D concentrations were quite high, indicating that participants may be using additional non-prescribed supplements.

Research indicates that physical activity, particularly vigorous exercise, is linked to higher 25(OH)D concentrations in middle-aged and older individuals [34–36], often due to increased sun exposure [37] but also through intrinsic effects on vitamin D metabolism [34, 36]. Exercise not only boosts serum 25(OH)D concentrations [38, 39] but may also enhance vitamin D receptor and CYP27B1 expression in skeletal muscle [40], suggesting a direct impact on vitamin D metabolism. In our study, middle-aged participants with higher activity levels had superior serum 25(OH)D compared to less active peers, although this association was not observed in older participants. This underscores the potential of physical activity to prevent vitamin D decline, especially in populations like Finnish middle-aged women who generally maintain adequate vitamin D status.

Research consistently shows obesity’s negative impact on 25(OH)D concentrations in middle-aged and older adults [36, 41]. A systematic review of 34 studies identified a slight but significant inverse correlation between BMI and serum 25(OH)D concentrations [42]. Various theories have been proposed, including reduced sunlight exposure among obese individuals [43] and lower vitamin D supplement usage compared to those of normal body mass [41]. Additionally, the hypothesis suggests that vitamin D is more rapidly depleted in obesity due to higher absorption by adipose tissue [44]. Our findings confirm that obese individuals in both middle-aged and older groups have significantly lower serum 25(OH)D concentrations, underscoring obesity as a key factor in diminished vitamin D levels.

Our study utilized a linear mixed model to explore the impact of vitamin D on muscle function and mass, controlling for confounders. Nonetheless, no significant association was found between 25(OH)D concentrations and muscle outcomes in middle-aged women, as well as older men and women. Previous research has documented the association between vitamin D status and muscle outcomes in older adults [15, 45, 46]. Therefore, the lack of a relationship between 25(OH)D concentrations and muscle mass and muscle function may be due in part to the generally high vitamin D levels of the subjects included in this study. Interestingly, in the current cohort, most of the subjects were, in addition to a fortified diet, either taking vitamin D as a supplement or as prescribed drugs. In a meta-analysis, vitamin D supplementation has been shown to protect against lean mass loss and improve lower extremity muscle strength in postmenopausal women [47]. However, other clinical studies have found no significant effect of vitamin D monotherapy on indices of sarcopenia such as muscle mass or strength in community-dwelling older adults [48]. Alternatively, there may be an optimum 25(OH)D concentration required to regulate protein metabolism in skeletal muscle, and exceeding it may not be effective. Further studies are warranted to investigate the optimal 25(OH)D concentration for the prevention of sarcopenia.

This study leverages the robustness of large population-based data, enhancing the generalizability of our findings. However, it is crucial to acknowledge limitations. First, the sampled participants likely represent a healthier segment of the population. This selection bias suggests we might not have fully captured those with the poorest health statuses and the lowest vitamin D status. Second, muscle quality and function were not assessed uniformly between cohorts, as DXA was used in the middle-aged cohort and BIA in the older cohort, which may limit comparability. Third, we did not account for self-prescribed vitamin D supplements in the older cohort, potentially affecting 25(OH)D concentrations. Lastly, physical activity was measured via self-reported questionnaires, which could introduce bias; objective measures like accelerometry would have provided more accurate data.

In conclusion, cohort data from middle-aged and older women and men were used to investigate the relationship between serum 25(OH)D concentrations and SMM and function during the menopausal transition and aging. Sufficient 25(OH)D concentrations were found in the majority of both Finnish middle-aged women and older men and women. However, while menopause leads to a decrease in muscle mass and function, maintaining adequate 25(OH)D concentrations alone does not prevent this decline in middle-aged women. Future studies should investigate the optimal serum 25(OH)D levels needed to affect muscle quality, function, and strength across different age groups, given the lack of significant association found in this study. Examining the influence of vitamin D-related factors, such as vitamin D receptor (VDR) polymorphisms, on muscle function could also reveal insights into individual variability in response to vitamin D fortification or supplementation.

## Supplementary information

Supplementary information

## Data Availability

Data analyzed during this study are included in this published article. The complete individual-level data is not publicly available due to privacy or ethical restrictions; however, data can be made available upon reasonable requests and subject to ethical approval. The metadata of the ERMA study (doi:10.17011/jyx/dataset/83491) and the AGNES study (doi.10.17011/jyx/dataset/83811) are publicly available at the JYX Digital Repository of the University of Jyväskylä.
