# Supplementary material for: Vitamin D sufficiency and its relationship with muscle health across the menopausal transition and aging: Finnish cohorts of middle-aged women and older women and men
Source: Eur J Clin Nutr. 2025 Apr 2;79(8):731–9. doi: 10.1038/s41430-025-01610-4 (PMC12353869; doi:10.1038/s41430-025-01610-4)
Supplement: Supplementary file 1 — Supplementary information [file 41430_2025_1610_MOESM1_ESM.docx]

Supplementary information

**Additional information regarding the physiological tests**

Participants were also advised not to consume caffeine or smoke for two hours before the test and to consume only light meals on the test day, preferably two hours before their arrival at the research center. The tests were carried out by trained research staff, who ensured the correct procedures were followed for each physical performance test. Participants were familiarized with the tests through practice trials before the actual measurement took place.

Maximal isometric knee extension force was assessed with participants seated on a custom dynamometer chair (Good Strength; Metitur Oy, Finland), with their knee positioned at a 60° angle from full extension. Participants were instructed to exert maximal effort while extending their knee. The peak force, recorded in Newtons (N), was used to calculate isometric muscle torque by multiplying it by the length of the lower leg, defined as the distance from the lateral knee joint line to the center of an ankle cuff.

Grip strength on the dominant hand was measured in Newtons with the elbow bent at 90° and the arm secured to the armrest of the dynamometer chair using a hand-held adjustable dynamometer (Good Strength; Metitur Oy, Finland). After a practice trial, participants performed 3 to 5 short maximal contractions, with 30 seconds of rest between each attempt. They were instructed to squeeze the handle with maximum effort, maintaining the grip for 2–3 seconds. The highest recorded value was used for analysis.

Lower body muscle power was evaluated using a countermovement jump performed on a contact mat (Ergojump, Junghans GMBH-Schramberg, Germany). This test measures the ability to rapidly raise the body’s center of gravity during a vertical jump. The height of the vertical jump, expressed in centimeters, was calculated based on flight time (t) using the formula: (g × t²) ÷ 8 × 100.
